# The state of health services partnering with consumers: evidence from an online survey of Australian health services

**DOI:** 10.1186/s12913-018-3433-y

**Published:** 2018-08-10

**Authors:** Jane Farmer, Christine Bigby, Hilary Davis, Karen Carlisle, Amanda Kenny, Richard Huysmans

**Affiliations:** 1 Social Innovation Research Institute, Swinburne University of Technology, John Street, Hawthorn, Melbourne, Australia; 2 Living with Disability Research Centre, La Trobe University, Bundoora, Melbourne, Australia; 3 Centre for Social Impact, Swinburne University of Technology, John Street, Hawthorn, Melbourne, Australia; 4 College of Medicine and Dentistry, James Cook University, Townsville, Australia; 5 Australian Institute of Tropical Health and Medicine, James Cook University, QLD, Townsville, Australia; 6 La Trobe Rural Health School, La Trobe University, Bendigo, Australia; 7 Raven Consulting Group, Unit 4, 62 Wattletree Road, Armadale Vic, Melbourne, 3143 Australia

## Abstract

**Background:**

Involving consumers in producing health services is mandated in many countries. Evidence indicates consumer partnerships lead to improved service design, quality and innovation. Involving participants from minority groups is crucial because poor understanding of distinctive needs affects individuals’ service experiences and outcomes. Few studies consider service compliance with consumer partnering requirements or inclusion of minority group participants.

**Methods:**

An online survey structured by domains of the Australian National Safety and Quality in Health Service Standards (NSQHS, 2013), was conducted. Questions covered consumer partnering in service planning, management and evaluation plus patient care design and inclusion of consumers from minority groups. Approximately 1200 Australian hospital and day surgery services were identified and 447 individual email addresses were identified for staff leading consumer partnerships. Quantitative data were analysed using SPSS. Qualitative responses, managed in NVivo, were analysed thematically. Frequencies were produced to indicate common activities and range of activities within question domains.

**Results:**

Comprehensive responses were received from 115 services (25.7%), including metropolitan and non-metropolitan, private and public service settings. Most respondents (95.6%) “partnered with consumers to develop or provide feedback on patient information”. Regarding inclusion of participants from minority groups, respondents were least likely to specifically include those from socially disadvantaged backgrounds (23.6%). Public health services were more likely than private services to engage with consumers.

**Conclusions:**

The survey is the first to include responses about consumer partnering from across Australia. While many respondents partner with consumers, it is clear that more easily-organised activity such as involvement in existing committees or commenting on patient information occurs more commonly than involvement in strategy or governance. This raises questions over whether strategic-level involvement is too difficult or unrealistic; or whether services simply lack tools. Minority views may be missed where there is a lack of specific action to include diversity. Future work might address why services choose the activities we found and probe emerging opportunities, such as using social media or online engagement.

**Electronic supplementary material:**

The online version of this article (10.1186/s12913-018-3433-y) contains supplementary material, which is available to authorized users.

## Background

Involving consumers in producing health services is mandated in many countries. Evidence indicates consumer partnerships lead to improved service design, quality and innovation [1].

Since the seminal 1978 Declaration of Alma Ata [2], it has become widely accepted that citizens have both rights and duties to participate in planning and implementing their health care and there has been a shift towards recognising clinicians, managers, consumers and carers as partners in the health system [3].

Participation of consumers from diverse backgrounds is critical in partnering with consumers as one way of ensuring health services understand the distinctive needs of minority groups [4]. While health services accreditation systems - e.g. in Australia, the National Safety and Quality in Health Service Standards (NSQHS) - require health organisations to involve consumers, [5] there have been few large-scale studies, across health systems, of how accreditation and standards affect consumer partnership activities. This is significant as grandiose aims for consumer involvement are suggested, including from informing day-to-day service provision (to improve services) through to roles in governance and driving strategy (implying a deep and wide relationship inside health agencies) [6, 7]. Our study considers the state of the art of consumer partnership as defined in the NSQHS standards [5] in Australia, in 2017. The standards cover all health service organisations that provide ‘hospital and day service procedures’ [see: www.safetyandquality.gov.au/our-work/assessment-to-the-nsqhs-standards/information-for-accrediting-agencies/] which includes ‘a wide variety of health service organisations…[of]…variable size, structure and complexity of health service delivery models’ ([8], p.3). The study comprised a survey of these Australian health services’ activities in partnering with consumers. In particular, we probed whether and how, health services partner with minority groups including people from diverse cultural and ethnic backgrounds, Indigenous Australians and people with disabilities.

Health services standards indicate what is prioritised in a health system and what is considered fundamental to consumer safety and quality of experience. In an overloaded system, standards signal to management and staff what must be done and not just what could be done. Inclusion of consumer partnering as a standard, therefore, highlights this as central rather than optional, to service delivery. Standard Two of the ten domain, Australian NSQHS [3] concerns partnering with consumers. It lists a range of activities in which consumers should be involved, including service planning, designing care, management and evaluation. The necessity to involve a diverse group of people from across the community is also highlighted so the needs of all potential service users can inform decision-making. Given the growing sophistication of advice about implementing consumer partnerships - for example, NHS England [7] has a new suite of evidence-based patient and public involvement guidance - we considered it timely to study how services across Australia partner with consumers and the barriers experienced. This evidence will identify weaknesses and areas requiring further development to be tackled by policymakers, practitioners and researchers. While our survey was of Australian services, findings are relevant internationally in highlighting challenges of embedding consumer partnership systemically and in identifying examples of good practice.

Consumer partnering covers ‘patients, families, carers and other support people’ ([5]; p.5); a group that might be considered ‘experts by experience’ ([7]; p.6). The concept of consumer partnering aligns with international movements [9], including English policy on patient and public involvement [5] and Canadian policy on including citizen views in health system and policy planning and decision-making [10]. Signalling the inherent dualistic nature of the relationship between services and citizens in these partnering activities, the Australian NSQHS standard states consumers: ‘have the right and duty to participate individually and collectively in the planning and implementation of their health care’ ([5] p.6).

Given the significance of consumer partnerships, health service managers must ensure they occur [11]. Gill and Gill [12] suggest that implementing consumer partnering in health can be challenging as it requires staff moving from positions of power to ‘involved egalitarian relationships’ with consumers, requiring that ‘professionals must relinquish control as consumers accept greater responsibility’. However, as Taylor ([13] pp.114–117) notes, ‘instrumental’ partnering instigated by managers and staff still leaves it a ‘top-down’ process essentially implemented to achieve service goals, in contrast with ‘bottom-up’ citizen activism.

An early evaluation of the Australian standard on consumer partnering found that ‘consulting with consumers to provide feedback on patient publications’ was considered the easiest way to partner by health service respondents [14]. The evaluation found that partnering was challenging, with involvement in governance considered particularly difficult. Understanding the purpose of partnering, obtaining executive endorsement, and strategies for implementation were also raised as problematical issues. The evaluation did not probe diversity of consumers involved – an area frequently raised as challenging in the literature [15].

NHS England produced guidance for health services to assist them with identifying strategies and resources for inclusion of different minority groups in consumer partnerships [16]. This was intended to move partnering from the traditional tendency to engage mainly from ‘snowy white peaks’ [17]. The English guidance summarises evidence about effective strategies for engaging with different minority groups or adapting general engagement to better include people from minority groups. De Freitas and Martin [4] highlight that inclusion of minority voices is important in exposing health practitioners to alternative understandings of health and healthcare that challenge dominant mainstream thinking. NHS England [16] defines minority groups as those protected by the Equality Act of 2010 [18], including those who experience inequality due to disability, race, ethnicity or sexual orientation. In our study we were particularly interested to probe strategies being used by Australian health services to partner with diverse community members. Henceforth, we use the term ‘minority groups’ to encompass a range of social groups recognized as experiencing exclusion.

As well as simply providing a range of perspectives, inclusion of minority voices is imperative for community health improvement. Minority groups, tend to have poorer health outcomes associated with environmental, socio-demographic or individual characteristics [19]. Examining causal pathways of health gaps, Trollor and colleagues [20] highlight that, for people with an intellectual disability, lack of knowledge and awareness within health systems and lack of advocacy underpins poor service adaptation for their needs. In turn, this reflects in the high rate of hospital readmissions and avoidable deaths for people with an intellectual disability. Similarly, Shimmin and colleagues [21] examined health service experiences of socio-economically disadvantaged Canadians, including people from Indigenous and migrant backgrounds. They raised, in particular, poor understanding within the health system of consumers who had experienced trauma, and the few adjustments to take account of those with trauma histories which influenced engagement with health services and compounded poor health status. Partnering with participants of minority groups and using their experiences to inform service provision, is therefore likely to produce significant benefits in improving services for all consumers. Affirmative action to include minority participants may also counterbalance the tendency of these groups not to complain [22], which can mean their health challenges are seldom raised, and often neglected.

Involvement in public meetings or committees tend to be ineffective strategies for including minority groups as they can be intimidating for those with low confidence or communication difficulties [23, 24]. Those who fear stigma are unlikely to volunteer in health partnering; for example, reticence of LGBTI people to come forward due to feelings of isolation, insecurity and low trust in health services has been found [25]. So, considerable effort needs to be invested in building a secure and positive health organisation context. Partnering can be operationally difficult, requiring alternative ways for participant involvement such as allocating specific time in meetings, allowing for skype or telephone participation, for supporters, peers or translators to be present or different ways of expression for people with communication challenges [16].

A strand in the literature warns health services against trying to include every possible type of consumer as no-one can be representative of their entire social group or defined by one set of characteristics [26]. Nonetheless, attracting members of minority groups whose health experiences are unlikely to be represented by others is crucial. Frawley and Bigby [23] suggest progress has been made to include people with a disability on boards, advisory committees and reference groups in health, but under-representation persists.

Our study explored strategies and activities health service managers deploy to partner with consumers by surveying a comprehensive population of Australian health services that provide hospital or day surgery services. We aimed to characterise partnering activities, identify how health services recruit consumers, and the extent to which minority groups were included. The research questions were 1) what is the state of consumer partnership in Australian health services? And, 2) to what extent do Australian health services make efforts to include minority groups in partnership? Using an online survey, we asked about activities described in the NSQHS Standard Two ‘Partnering with Consumers’ [3]. Australian hospital and day surgery services are all accredited against these and thus they indicate the ‘gold standard’ of what health services should be enacting. This study obtained ethical approval from the La Trobe University Human Research Ethics Committee (S16–129).

## Methods

We designed and applied an online questionnaire, targeted at hospital and day surgery services, that asked a set of questions covering all of the activities for consumer partnering suggested in the Australian NSQHS standards [5]. These are structured in three domains in the standards: service planning; patient care design; and service management and evaluation. The standards also note the significance of including people from diverse backgrounds so we included a set of questions about inclusion of minority groups [16]. The questionnaire text is available as an Additional file 1. The questionnaire was built using Qualtrics (https://www.qualtrics.com/), and was targeted at Chief Executive Officers (CEOs) of health services or other staff members responsible for service quality and safety accreditation. Replies to the questionnaire were anonymous. This form of survey is a cost-effective way of reaching respondents scattered across a wide geographic area. Closed questions provided a pre-defined set of responses plus there was space for comments. Response rates to online surveys have tended to be lower than for postal surveys [27] but we pre-identified participants, so we aimed for a 25% response rate overall, which was met.

### Sample

We took a whole of population approach and used the Australian Hospitals and Aged Care Database to identify services (see http://jpmmedia.com.au/direct-marketing/hospitals-aged-care-database/about-hospitals-aged-care-database/). To ensure questionnaires were directly sent to the most suitable participant, we telephoned all health organisations to identify an appropriate personal, rather than general organisation, email address. Criterion for inclusion was that the participant was either a) the CEO or equivalent of a participating health service, b) CEO representative (nominated by the CEO), or c) the person in charge of accreditation (and the NSQHS Standard Two specifically) for the particular health service. One participant/return only, from each emailed address was requested to avoid multiple responses from the same service. While compiling the sample, we found some CEOs have responsibility for multiple health service organisations. We noted these in our database and decided to send each of these CEOs only one email. This was to avoid confusion about distinguishing between health services when surveys were returned. After deducting duplicates, health services that had closed or merged, and those covered by a ‘parent’ health service organisation responsible for accreditation, we identified a total of 447 individual participant email addresses, covering 1200 health services in total.

### Data collection

The survey (see Additional file 1) was designed to collect data to address the two research questions. Demographic data, including about the state or territory location, respondent job title, and service type, were collected. For type we devised a classification aligned with Australian health services that provide hospital or day surgery services: i.e. publicly-funded, publicly-funded community health service, metropolitan, regional, sub-regional, small rural, multi-purpose, community-based, private hospital or other (asking respondents to specify). We devised this typology based on testing of survey drafts with four health service managers. Australian states have different categorisations for health service types so we devised a typology intended to be useable across Australia.

Questions to address Research Question 1 followed the activities for consumer partnering identified within domains of NSQHS Standard Two; for example, within the domain ‘service management and evaluation’, questions included: “Have you used consumers in the analysis of safety and quality performance information and data, to develop plans for your health service?” and “Have you partnered with consumers in the analysis of patient feedback data?”. Responses could be: yes, no or don’t know, followed by a request for explanation (e.g. briefly list how you have done this). To address Research Question 2, we included a set of minority groups likely to have particular needs or perspectives based on their experiences. We based these on categories from a NHS England document [16]. We asked respondents if their services had “specifically sought to include” each of the specified minority groups. The survey asked how health services recruited consumers from these groups; or if not, then why. Finally, a box was provided where respondents could record anything else on the topic that they wanted to say.

Survey questions and format were loaded into Qualtrics. A link to the survey was sent by email to the identified participants. The email included an introduction letter, plain language information sheet and ethics statements. Once the plain language statement was read, the survey took about 15 min to complete. Consent was obtained via response to the survey. All surveys were returned anonymously via Qualtrics. The survey was first emailed in September 2016 (69 surveys completed), with follow-up reminders in October 2016 (43); and two in November 2016 (33 and seven). We undertook to send all participants a findings summary at study end.

### Data analysis

Quantitative survey data were input into SPSS (IBM Version 23) and exploratory analysis conducted to examine respondent characteristics and frequencies of responses. Simple cross tabulations and Chi-square tests were calculated to determine associations between health service type (private versus public) and geographic location (metropolitan versus non-metropolitan) and service engagement in consumer partnership activities. Qualitative responses were input into NVivo (Version 11). These tended to be brief and note-like rather than discursive, due to the nature of the survey response format requested (i.e. ‘please list…’). Inductive analysis was conducted to identify thematic categories. Data were then allocated to thematic categories and frequencies of quotes on themes counted [28]. KC conducted initial qualitative analyses. The other authors also read the data. JF checked and verified the analysis.

## Results

A total of 447 participants, each responding for one or more health services were contacted to participate. Responses were returned by 152, giving a 34% response rate. Thirty-seven surveys were excluded from analysis due to responding only to Section 1: demographic data. Thus, the final number of complete, eligible survey responses was 115 (25.7% response rate). As Table 1 shows, the largest proportion of these indicated their service was located in a metropolitan area (54.8%), and proportions of respondents from private and public health services were similar, 46.9% private and 52.1% public.

**Table 1:** Characteristics of respondents

Characteristics of health service of respondent	N in sample	N of respondents (%)
Location of service		
Metro	283	63 (54.8)
Non-Metro	164	46 (40.0)
Missing or unsure	–	6
Total	447	115
Type of service		
Public	172	60 (52.1)
Private	275	54 (46.9)
Missing	–	1
Total	447	115
Response by state		
Australian Capital Territory	7	2 (1.7)
New South Wales	126	27 (23.5)
Northern Territories	5	2 (1.7)
South Australia	52	8 (7)
Tasmania	12	3 (2.6)
Queensland	66	14 (12.2)
Victoria	151	50 (43.5)
Western Australia	28	9 (7.8)
Total	447	115

### Consumer partnering activities

Table 2 shows the number of all health services that stated they were engaged in the consumer partnering activities covered in the questionnaire (i.e. that responded yes – their service has undertaken this activity). Responses are broken down by type (private and public) and geographic location (metropolitan and non-metropolitan). Due to the nature of the sample, none of the health services are specifically primary care services, although some of the services may provide primary care as part of their service portfolio. There are no specific Aboriginal and Torres Strait Islander hospital or day surgery services, but some hospitals responded that we can infer would have high use by the Aboriginal and Torres Strait Islander community.

**Table 2:** Number of respondents of health services that reported engagement in consumer partnering activities

Activity	Total number of services responded yes (%)	Breakdown by type of service			Breakdown by location		
		N Private services responded yes (%)	N Public services responded yes (%)	Chi-square test of association	N Metro services responded yes (%)	N Non-Metro service responded yes (%)	Chi-square test of association
Consumer partnering in service planning							
To develop or provide feedback on patient information	109 (95.6)	53 (96.4)	56 (94.9)	Not calculated^a^	61 (96.8)	39 (95.1)	Not calculated^a^
In quality improvement activities	94 (84.7)	47 (88.7)	47 (81.0)	1.25 *p* = .264	54 (90.0)	32 (78.0)	2.97 *p* = .085
In decision making about safety and quality	91 (79.1)	42 (76.4)	49 (81.7)	0.49 *p* = .485	49 (77.8)	35 (83.3)	0.38 *p* = .535
Provided training in partnering with health service	86 (76.1)	37 (69.8)	49 (81.7)	2.17 *p* = .140	46 (75.4)	32 (76.2)	0.01 *p* = .936
In strategic and operational services planning	78 (69.3)	27 (50.0)	52 (86.7)	**17.96** ***p*** **< .000**	39 (62.9)	31 (73.8)	1.46 *p* = .227
Governance structures to facilitate partnering	78 (68.4)	31 (57.4)	47 (78.3)	**5.76** ***p*** **= .016**	40 (63.5)	29 (70.7)	1.61 *p* = .281
Consumer partnering in designing care							
Implemented training for staff about consumer partnering	89 (78.1)	41 (75.9)	48 (80)	0.28 *p* = .600	49 (77.8)	34 (81.0)	0.11 *p* = .737
Used consumers in service design	77 (67.5)	31 (57.4)	46 (76.7)	**4.81** ***p*** **= .028**	44 (69.8)	29 (69.0)	0.07 *p* = .785
Involved consumers in training the clinical workforce	72 (63.2)	31 (57.4)	41 (68.3)	1.45 *p* = .227	43 (68.3)	24 (57.1)	1.57 *p* = .209
Consumer partnering in service management & evaluation							
Analysis of patient feedback data	81 (73.0)	34 (65.4)	47 (79.7)	2.86 *p* = .091	42 (66.7)	36 (85.7)	**4.57** ***p*** **= .033**
Analysis of safety & quality performance information & data	73 (65.8)	29 (55.8)	44 (74.6)	**4.34** ***p*** **= .037**	44 (69.8)	26 (61.9)	0.55 *p* = .456
Develop information about the health service safety & quality performance	68 (61.3)	28 (53.8)	40 (67.8)	2.27 *p* = .132	39 (61.9)	27 (64.3)	0.01 *p* = .904

Percentages for each survey item were calculated as valid percentage i.e. missing values were excluded

^a^Chi squared calculated only if all expected cell frequencies are ≥5

Exploratory descriptive analysis showed that, overall, the majority of respondents reported their health services were engaged in partnering activities. The proportions of services engaged in these activities ranged from 95.6% of respondents partnering with consumers ‘to develop or provide feedback on patient information’, to 61.3% partnering with consumers ‘to develop information about health service safety and quality performance’. Chi-square tests of association showed a significant association between type of service - public - and engagement in: strategic and operational services planning, governance structures, service design and analysis of safety and quality performance data (in bold see Table 2). Chi-square tests were calculated to determine if there was an association between metropolitan or non-metropolitan location, and partnering activities. Analysis showed only one significant association between location of service – non-metropolitan - and engagement of consumers in analysis of patient feedback data (in bold see Table 2).

### Partnering involving minority groups

The survey asked about inclusion of people from minority groups in consumer partnering. Descriptive analysis indicated that specific steps to include the minority groups listed occurred for less than half of responding services (see Table 3). People from different cultural and ethnic backgrounds was the most frequently reported group that services sought to include (45.5%). Socially disadvantaged people was the least reported group (23.6%). Responses are broken-down by type (private and public) and geographic location (metropolitan and non-metropolitan). Chi-square tests showed a significant association between service type - public - and services seeking to include all of the minority groups described, except people of different genders (in bold Table 3). There was a significant association between location – metropolitan – and seeking to include people from different cultural and ethnic backgrounds and people from the LGBTI community (in bold Table 3).

**Table 3:** Number of respondents of health services that reported seeking to include particular groups in consumer partnering

Particular social groups health services sought to include in consumer partnering	Total number of services responded yes (%)	Breakdown by type of service			Breakdown by location		
		N Private services responded yes (%)	N Public services responded yes (%)	Chi-square test of association	N Metro services responded yes (%)	N Non-Metro service responded yes (%)	Chi-square test of association
People of different cultural and ethnic backgrounds	50 (45.5)	18 (34.6)	32 (55.2)	**4.67*p*** **= .031**	34 (54.0)	14 (33.3)	**3.94** ***p*** **= .047**
Indigenous Australians	45 (41.3)	11 (21.6)	34 (58.6)	**15.37*p*** **< .000**	28 (45.2)	16 (38.1)	0.71 *p* = .392
People with mental health or psycho-social issues	42 (38.9)	12 (23.5)	30 (52.6)	**9.59** ***p*** **= .002**	25 (41.0)	14 (33.3)	0.17 *p* = .673
People with different levels of physical or sensory disabilities	41 (38.0)	8 (15.4)	33 (58.9)	**21.71*p*** **< .000**	23 (37.1)	17 (41.5)	0.09 *p* = .760
People with cognitive disabilities unrelated to age	30 (28.3)	8 (16.3)	22 (38.6)	**6.44** ***p*** **= .011**	17 (28.3)	12 (29.3)	0.01 *p* = .950
People of different sexual orientation, including LGBTI	28 (25.7)	12 (23.5)	16 (27.6)	0.23 *p* = .629	21 (33.9)	6 (14.3)	**4.78** ***p*** **= .029**
Socially disadvantaged people	26 (23.6)	2 (3.8)	24 (41.4)	**21.39*p*** **< .000**	14 (22.2)	10 (23.8)	0.22 *p* = .640

Percentages for each survey item were calculated as valid percentage i.e. missing values were excluded

### Qualitative responses: partnering activities

As noted previously, due to the nature of the questionnaire format (where participants were asked to ‘list’, for example, activities or reasons for doing/not doing, activities), qualitative responses tended to short, note format, rather than discursive format. This type of response lent itself to compiling frequencies of responses by theme, rather than writing summaries of each theme. Table 4 thus provides frequencies of partnering mechanisms reported (see Table 4); and Table 5 descriptions of partnering activities, along with verbatim exemplar illustrations of items listed.

**Table 4:** Frequency of reported mechanisms used by health services for consumer partnering

Mechanisms for partnering (based on wording used by respondents)	N of responses to all survey questions on consumer partnering^a^
Community advisory committee	146
Representative at other committees	124
Surveys/interviews	50
Consumer focus groups	35
Via patient feedback	20
Representative at board	14
Via consumer groups	14
Presented/discussed at other committees	13
Reports to other committees and/or board	11
Policy documentation	7
Regular meetings with consumers	7
Via open forum	5
Discussed at meetings	4
Total number of free text responses	450

^a^Number of qualitative responses indicates the frequency of each theme across nine questions related to consumer partnering activities. Some services were engaged in more than one activity therefore responses from services were coded multiple times

**Table 5:** Frequency of comments describing partnering activities used by health services

Partnering activities	Number of free text responses	Example of comments
Involved in reviewing and giving feedback	157	- Consumer representative reviews our patient feedback surveys and makes recommendations - Consumer partnership committee reviews and comments on quality performance data and incident management. - Review of clinical indicators and feedback on some strategies that could be used to improve outcomes. - Review of our acute health handbook. - Reviews our patient information brochures for health literacy.
Involved in service design	40	- Redeveloping the site in the next 2 years and consumers have been involved in a number of service delivery projects. - Consumers involved in priority service design projects (e.g. Outpatients Transformation, Nightlife, Culture Transformation).
Involved in planning (including the types of activities)	35	- Consumers on Board involved in service planning. - Consumers are invited to participate in strategic planning days and workshops with all CEO Executive and Dept. heads present, they are asked for input and comment. Some suggestions are adopted into current strategic plan and highlight areas requiring work.
Contributed to the design of information/ brochures	31	- Consumer committee review and advise on patient information brochures. - Consumers have been used to design the Quality of Care report and provide feedback on the website.
Involving consumers in audits	19	- Involved in audit of improvement activities such as the patient and carer escalation project. - Consumers audit some clinical data. - Involved in bedside patient experience audits.
Involved in decision making	11	- Assisting in the design and selection of new patient and visitor furniture, soft furnishings and décor. - We have 6 co- design improvement projects running with consumer input. - Involved in review of hospital signage, patient menu testing, implementing medication management plan, creating patient information videos for website.
Quality improvement activities	8	- Included on quality projects - part of project team memberships. - Monthly clinical audits, monitoring aspects of Excellent Care surveys monthly, regular clinical safety and quality focused executive rounding is accompanied by consumer advisors.
Total number of free text responses	301	

The most common mechanisms listed were community advisory committees and consumer representation on other health service committees. The most common activities of community advisory committees were reviewing and providing feedback on patient data, safety and quality data and patient information. A small number of comments indicated that some health services included their advisory committee in service decision-making. The committee that consumers were most likely to attend as representatives was ‘quality and risk’/ ‘quality and safety’.

### Qualitative responses: inclusion of minority groups

There were 160 qualitative responses to questions about including people from minority groups. Table 6 summarises the ways respondents “sought to include” participants, with illustrative comments. Some respondents noted more than one strategy. The most frequently reported active recruitment method was through other groups or services in the community. Other ways included approaching service users directly and through specific health service teams.

**Table 6:** Recruitment methods to include minority groups, reported by health services

Active recruitment	Number	Example comments
Through other groups/services	36	- Actively sought out local disability groups in community. - We used local multicultural organisations and local Indigenous organisations to assist in providing representatives on the consumer committee.
Approached service users	20	- We approached individuals in this category that have been patients at the hospital. - We approached individuals who had been patients at the hospital/family of individuals that have been patients at the hospital.
Through service staff/teams	17	- Cultural and linguistic diversity team in collaboration with our consumer engagement team do this at our various facilities. - We made contact with the Traditional owners and Elders via the hospital liaison team. We then had a morning tea to introduce the Elders to the Chief Executive.
Community advisory committee	13	- We are a very diverse organization. Our community advisory committee has representation from key community groups.
Targeted recruitment/ advertising	9	- Yes, again aim to increase this input. Posters across organisation advertising consumer register, advertised in external newspapers. - Advertise in ethnic media.
Approached carers	6	- Our Cognitive Impairment committee has sought the involvement of a carer of an individual with dementia.
Governance structures	2	- Aboriginal Governance Committee is a Board sub-committee with Chair and representation from the Aboriginal community.

### Reasons for not seeking inclusion of minority groups

Table 7 summarises the reasons given by respondents about why they did not seek inclusion of minority groups. A total of 276 responses were received. Some gave more than one reason. Some respondents considered it irrelevant or inappropriate for their organisation, while others suggested there are low numbers of people from minority groups in the local area, that they have limited resources to seek out participants, or there was a lack of interest from relevant groups.

**Table 7:** Reasons why health services did not seek inclusion from minority groups

Reasons why services did not seek inclusion	Number	Example comments
Not appropriate /relevant	79	- Minimal relevance to our services. - Not really relevant at the moment but if demand indicated we would look at it.
Limited diversity	61	- Limited diversity amongst patient group. - People from diverse backgrounds form a very small part of our patient population. Their needs are considered in different ways and on an individual basis.
All welcome	42	- We do not have a very culturally diverse demographic so while no one is excluded we haven’t focused on including any specific groups. - Because everyone is welcomed regardless.
Planned in future	24	- Very difficult in our geographical area and the work that we undertake. However there is plan to undertake in the next 12 months. - Beginning to consider a strategy for this. No funding at this stage - challenges to identify a feasible approach to targeted consultation.
Current methods unsuccessful	23	- Difficulty recruiting members for the committee. Have looked at cultural and ethnic clusters within our region and have been unable to recruit anyone to date that is not white Caucasian Australian. - Difficulties in a small community but keep trying.
Committee self nominates	15	- Committee members (consumers or carers) are self-nominating.
Lack of resources	14	- Lack the resources to specifically involve particular members of the community. - Although we do have engagement with Indigenous communities, there is limited engagement with other communities due to lack of an engagement framework and resources.
Identification challenge^a^	14	- No process for identifying or communicating with the LGBTI community. - Not on purpose, we don’t ask the sexual orientation of our patients.
Lack of community interest	4	- No interest from consumers to participate other than on the day of their appointment. - Limited number of people interested in roles in small rural health service & ageing population.

^a^Mostly in relation to question about the LGBTI community

## Discussion

This is the first Australian survey of health services seeking information about partnering with consumers and efforts to include participants from minority groups. The NSQHS Standard Two requires that all Australian health organisations providing hospital or day surgery services partner with consumers and services undergo a regular accreditation process [3]. Given this imperative to partner, it might be assumed that all health services targeted would be making efforts in this domain.

Three key issues emerged: 1) while most services are partnering with consumers in various activities, the highest proportion of involvement appears to be through engagement in relatively operational or ‘bolt-on’ activities (e.g. advising on patient information or involvement in a specific consumer committee), rather than through apparently more systemic approaches (e.g. involving consumers in service design and workforce training activities) or activities affecting service strategy (e.g. strategic planning or governance); 2) there appears to be limited activity around inclusion of minority groups; 3) there appear to be differences in the activities of public versus private, and non-metropolitan versus metropolitan, services. These issues are discussed below.

### Most services are partnering in several activities – mostly using committees

The majority of services are engaged in partnership with consumers (95%) and used community advisory committees as a key mechanism. One of the requirements of the NSQHS [3] in relation to consumer partnership is ‘establishing governance structures’. Forming a specific community advisory committee appears to be a relatively non-disruptive way for services to meet this requirement, by ‘bolting-on’ a new committee rather than taking steps to integrate consumers into existing committee structures and activities. This finding aligns with previous findings of the Australian Commission on Safety and Quality in Health Care [14] evaluation where involving consumers in committees was considered, by health services, to be one of the easiest consumer partnership activities.

Through involvement in a specific consumer advisory committee or being placed as a consumer representative on other committees, our findings show that consumers are drawn into various activities. However, these could be interpreted as being in relatively superficial areas of activity - such as providing feedback on patient information. There are different ways to view this situation. Established frameworks for analysing citizen involvement might understand this activity as ‘consulting’ [29] or ‘participation as a means’ [30] and thus relatively tokenistic and instrumental; with deeper structural and more system-wide involvement necessary to producing the empowered citizens and communities that bring population health impacts [31].

However, Tambuyzer and colleagues [32] highlight that, in consumer partnering, ‘one size does not fit all’ [p145]. Participation does not always have to aim for complex activities and there should be awareness that consumers may not desire to be involved in service strategy formulation or governance. It may boost consumers’ confidence to work together as a group, rather than be spread as individuals across a range of activities. Tritter and McCallum [33] highlight that consumer partnering in health activities is a complex business – that different individuals will want to contribute in different ways – and that health organisations are best served if citizens provide a range of inputs from commentary to strategic and directional involvement.

This makes having a specific consumer committee a beneficial activity, but perhaps within a portfolio of optional activities for partnering. If a consumer committee is the main focus of activity, then this runs the risk of appearing to be an easily-established after-thought, bolted-onto rather than integrated into a health service organisation. O’Shea et al. [34] suggest that, if consumer partnering is about co-production of services rather than ‘box-ticking’ to say a consumer has been consulted, then health services should include consumers in a range of activities where their perspective is of equal value to health employees [34].

### Inclusion of minority groups is reported less

Just under half of responding services had recruited consumer participants from minority groups. Inclusion of diverse participants is often highlighted as challenging in consumer partnering [35]. Nonetheless, we found several health services actively applying strategies to include minorities, including approaching other groups and services, their own service users and even targeted advertising. This shows that inclusion of minority groups is an issue acknowledged as significant by some services that have implemented strategies to specifically attract minority groups. These services may be early adopters, providing useful experience from which others could learn. By contrast, some respondents noted that participants from minority groups were not actively sought out because they already attained diversity due to the demographics of their location.

Some service respondents said they did not recruit from minority groups because it was irrelevant. Perhaps this is justifiable for some specialised services or for services located in areas with homogeneous populations. However, given research showing that minority groups tend to be under-represented in consumer partnering [36], it seems important for services to have robust evidence justifying non-inclusion of minority group participants. Other respondents highlighted difficulties in attracting minority group participants; for example, people from the LGBTI community in rural areas. This highlights a challenge in identifying participants with particular experiences without causing stigmatisation – an issue previously highlighted by other studies (e.g. [37]). Overall, it appeared that compared to other minority groups, less efforts were made to include socio-economically disadvantaged people. This could be because it is difficult to ‘label’ and specifically identify socially disadvantaged people – for example, in advertising – because they are perceived to have more ambiguous distinguishing characteristics, compared with disability or ethnicity. That is, perhaps it is more culturally embedded in Australia, to identify (i.e. label as ‘other’) some minority groups, compared to others. There was less inclusion of minority groups in non-metropolitan areas, and this could be due to reticence to look for, identify and include minority group participants due to the close-knit nature of rural places [38].

One issue may be a lack of tools to inform Australian health services about strategies for inclusion of minority groups, including in different geographical settings where there may be fewer numbers of people belonging to minority groups. Technology is increasingly mooted as potentially useful for including diverse views – either to actively include diverse participants in conversations (e.g. using social media discussions); or establishing specialist discussion groups that can be followed on social media to engage with important topics [39]. The NHS England [16] guidance on engaging with minority groups, notes that giving multiple ways to partner is useful for engaging minority groups, with technology adoption as one useful option. However, considerable care has to be taken with considering technology as a solution because digital exclusion due to poverty, variable connectivity, illiteracy, communication challenges and limited cultural sensitivity still present considerable barriers [39]. Using technology did not arise at all as a strategy deployed by health services in our survey, which may suggest a gap for future study.

### Differences between types of services

There was a significant association between public, as opposed to private, health services and consumer partnering activities; and a strong association between public health services and engagement with minority groups. This could be attributable to public services having to be more accountable for meeting public needs, as they are paid for by taxpayers. There is also evidence, particularly for small, non-metropolitan public health services, that they take considerable steps to build community inclusion and cohesion – so they are perhaps more culturally aligned with seeking inclusion of minority groups [40]. It could also be the case that public health services are more location-specific, embedded in the geographical communities, whose populations form strong connections to them. Private services tend to have wider reach, based more on volume of demand and patients’ capacity to pay than proximity to community, potentially making it more difficult to attract volunteer consumer partners.

Non-metropolitan services were more engaged in consumer partnering than metropolitan services, but were less likely to include minority groups. Greater engagement in consumer partnering activity may reflect a closer alignment with principles of primary health care provision among rural health services. Primary care strongly emphasises the role of community participation and empowerment [30] and some studies of Australian rural health services have highlighted a specific mission regarding community capacity-building [41]. Less inclusion of people from minority groups potentially reflects the demographics of Australian rural areas which are traditionally less culturally diverse than metropolitan Australia [42]. The challenges of highlighting ‘other-ness’ in rural places, for the potential stigma it brings, has already been noted above. It is perhaps more risky to try to identify diverse people in rural areas, more risky for them to be engaged, and due to smaller overall numbers, there are likely to be fewer participants from each of the minority groups.

### Study limitations

The study has limitations. There was uneven response across States and Territories, with highest numbers of responses from the States of Victoria and New South Wales. In relation to numbers distributed, there was poorest response from South Australia with only 15% responding compared to a range of 21–33% for most other States/Territories. The uneven response means we know little about how the culture of State health service systems might influence consumer partnering.

The survey was limited to services providing hospital and day surgery services because these services are covered by the national standards. If primary health services and Aboriginal and Torres Strait Islander services were also specifically studied, there is potential that a range of different strategies and activities would be identified. A range of strategies and deep involvement of consumers were found by Freeman et al. [30] in a study of six Australian primary healthcare services, for example.

We restricted response options to ‘yes’, ‘no’ or ‘don’t know’. It might have been useful to provide a finer-grained scale so that we could establish the extent to which activities were being conducted, although some information was provided through the listing of activities by services – where they took the time to do this. Providing a scale or more nuance around explaining activities could be useful in future research of this topic.

Findings might have limited transferability beyond the responding group. It is known that people are more likely to respond to surveys if the topic interests them (i.e. selection bias) - either because they are particularly affected by, or interested in, the items asked about. As people who respond almost certainly have different characteristics than those who do not, the results may be biased [43]. If the respondents are those that are most active and interested, then the findings could inflate the extent to which consumer partnering occurs. Innovators in consumer partnering may have self-selected to respond. Participants self-reported and the survey only accounts for CEOs’ (or their representatives’) perspectives. It is also possible that findings may be subject to response bias – given that the survey concerned compliance with standards, it is possible that respondents’ subjective reports were more positive than an objective measurement would verify. Further research also considering perspectives of consumers and staff, at the same services as the CEOs, would be valuable in generating a more accurate picture.

Finally, a revised set of Australian NSQHS standards has just been published [44]. Regarding consumer partnering, these highlight consumer committees and consumer involvement in activities around health literacy as core partnering activities. They continue to highlight the importance of including minority groups. While in the aforementioned respects, the new standards appear sharper than previously, there remains a lack of clarity about the nature of partnering that health services should pursue beyond apparently easier to bolt-on, activities.

Given the limitations outlined, as an exploratory study - systematically examining for the first time, the extent of consumer partnering in Australian health organisations – we suggest the findings make a significant and novel contribution to the field.

## Conclusions

This study represents a significant new contribution to the international literature on consumer partnering, for research and practice communities, as it quantifies partnering strategies in use in one developed country setting. Our findings enable identification of strategies, perceived best practice and highlight difficulties in implementing partnering. In particular, we present novel findings about strategies to include participants from minority groups. While study findings are from Australia, they are significant for informing international policy and practice about the strategies, and key remaining challenges, for hospital services partnering with consumers.

We found many services implementing strategies for consumer partnering that were easily ‘bolted-on’ to existing structures rather than integrated into the service, while others were employing partnering for more strategic aspects and making efforts to include minority groups. These latter services provide examples of good practice models and strategies that could be used by other services. Future research should also consider consumer and healthcare staff perspectives and probe the potential of digital participation as one method for consumer partnering, including use of social media. Examining participation using digital technologies seems a gap as no services in our study mentioned using digital participation.

## Additional file

Additional file 1: Health Services Partnering with Consumers. (DOCX 16 kb)

## Abbreviations

ACSQHC: Australian Commission on Safety and Quality in Health Care
CEO: Chief Executive Officer
LGBTI: Lesbian, gay, bisexual, trans and intersex
NHS: National Health Service
NSQHS: National Safety and Quality Health Service
UK: United Kingdom
WHO: World Health Organisation
