# Supplementary material for: The state of health services partnering with consumers: evidence from an online survey of Australian health services
Source: BMC Health Serv Res. 2018 Aug 10;18:628. doi: 10.1186/s12913-018-3433-y (PMC6086055; doi:10.1186/s12913-018-3433-y)
Supplement: Supplementary file 1 — Health Services Partnering with Consumers. (DOCX 16 kb) [file 12913_2018_3433_MOESM1_ESM.docx]

Health Services Partnering with Consumers

QID2 Through this 15 minute survey we hope to understand how health services are currently partnering with consumers, and how these partnerships are impacting on health care and health outcomes.Currently, health policy requires that health services partner with consumers, including clients/patients, carers and the public. However, there is little research that examines the partnership specifics and the associated impact on health care quality and health outcomes. This survey - intended for completion by CEOs (or their representatives) - is part of a larger program of research to inform policy and practice about the best ways to partner with consumers for optimal health care quality and health outcomes.The information you provide will be treated confidentially. We cannot trace individual responses and only the research team will see your completed survey. This project has ethical approval from the La Trobe University Human Research Ethics Committee (project number S16-129). The Ethics Committee identified no risks in completing this survey. We will send you a summary of findings to thank you for your involvement. Even if your health service does not engage in consumer partnerships, we would still be grateful if you would complete this survey.

QID7 The following questions ask some basic information about the type of health service.

QID5 What state(s) or territory(s) does your service operate in?

- ACT (1)
- NSW (2)
- NT (3)
- SA (4)
- TAS (5)
- QLD (6)
- VIC (7)
- WA (8)
- Other (please specify) (9) ____________________

QID8 What is your job title?

- CEO or equivalent (1)
- CEO delegate (please give job title) (2) ____________________
- Other (please give job title) (3) ____________________

QID9 What is the type of health service?

- Publicly funded health service (1)
- Publicly funded community health service (2)
- Metro health service (3)
- Regional health service (4)
- Sub-regional (5)
- Small rural (6)
- Multi-Purpose Service (7)
- Community-based service (8)
- Private hospital (9)
- Other (please specify) (10) ____________________

QID10 The following questions relate to how your service has partnered with consumers (including but not limited to current patients) in planning service activities. We are only concerned with 2013 to present.

QID11 Has your health service used governance structures to facilitate partnerships with consumers?  (e.g. consumers are involved on the health service board specifically to represent consumers/consumer groups)

- Yes (please briefly list how you have done this, the box can expand to fit your answer) (1) ____________________
- No (2)
- Don't know (3)

QID12 Has your health service used consumer partnerships in strategic and operational services planning? (e.g. involvement in drawing up the health service strategic plan)

- Yes (please briefly list how you have done this, the box can expand to fit your answer) (1) ____________________
- No (2)
- Don't know (3)

QID13 Has your health service used consumer partnerships in decision making about safety and quality initiatives? (e.g. consumers are actively involved in decision-making about safety and quality such as including consumers on a specific quality/safety of care committee)

- Yes (please briefly list how you have done this, the box can expand to fit your answer) (1) ____________________
- No (2)
- Don't know (3)

QID15 Has your health service used consumer partnerships in quality improvement activities? (e.g. involved consumers in safety / quality audits)

- Yes (please briefly list how you have done this, the box can expand to fit your answer) (1) ____________________
- No (2)
- Don't know (3)

QID16 Has your health service provided consumers with relevant orientation and training in partnering with your health service? (e.g. provision of a training course, induction pack, sent consumers on a course elsewhere)

- Yes (please briefly list how you have done this, the box can expand to fit your answer) (1) ____________________
- No (2)
- Don't know (3)

QID17 Has your health service used consumers to develop or provide feedback on patient information distributed by the organisation? (e.g. consumers and/or carers provide feedback on patient information publications prepared by the health service for distribution to patients)

- Yes (please briefly list how you have done this, the box can expand to fit your answer) (1) ____________________
- No (2)
- Don't know (3)

QID18 The following questions relate to how your service has partnered with consumers (including but not limited to current patients) in designing patient care. We are only concerned with 2013 to present.

QID20 Has your institution used consumers in service design? (e.g. in making decisions about the design or redesign of health services to better meet patient needs and preferences)

- Yes (please briefly list how you have done this, the box can expand to fit your answer) (1) ____________________
- No (2)
- Don't know (3)

QID21 Has your health service implemented training for clinical leaders, senior management or the general workforce about partnering with consumers? (e.g. workforce access training on consumer partnership)

- Yes (please briefly list how you have done this, the box can expand to fit your answer) (1) ____________________
- No (2)
- Don't know (3)

QID22 Has your health service involved consumers in training the clinical workforce? (e.g. consumers are involved in training the workforce)

- Yes (please briefly list how you have done this, the box can expand to fit your answer) (1) ____________________
- No (2)
- Don't know (3)

QID23 The following questions relate to how your service has partnered with consumers (including but not limited to current patients) in service management and evaluation. We are only concerned with 2013 to present.

QID24 Has your health service used consumers to develop information about the health service safety and quality performance, in a format that can be understood and interpreted independently by different audiences? (e.g. consumers involved in designing a Quality of Care report, website, blog etc. )

- Yes (please briefly list how you have done this, the box can expand to fit your answer) (1) ____________________
- No (2)
- Don't know (3)

QID25 Have you used consumers in the analysis of safety and quality performance information and data, to develop plans for your health service? (e.g. consumers are involved in a specific exercise or committee that considers data on quality of care)

- Yes (please briefly list how you have done this, the box can expand to fit your answer) (1) ____________________
- No (2)
- Don't know (3)

QID26 Have you partnered with consumers in the analysis of patient feedback data? (e.g. consumers participate in the evaluation of patient feedback data)

- Yes (please briefly list how you have done this, the box can expand to fit your answer) (1) ____________________
- No (2)
- Don't know (3)

QID27 The following questions relate to how your service has partnered with consumers (including but not limited to current patients) in general and the diversity of their participation/the participants. We are only concerned with 2013 to present.

QID28 Have you specifically sought to include people of different genders, including from the Lesbian Gay Bi-sexual Transgender and Intersex community, in consumer partnerships at your health service?

- Yes (how did you recruit participants?, the box will expand to fit your answer) (1) ____________________
- No (why not? e.g. too difficult; not relevant to the services we offer. The box will expand to fit your answer) (2) ____________________
- Don't know (3)

QID29 Have you specifically sought to include people from different cultural and ethnic backgrounds in consumer partnerships with your health service?

- Yes (how did you recruit participants?, the box will expand to fit your answer) (1) ____________________
- No (why not? e.g. too difficult; not relevant to the services we offer. The box will expand to fit your answer) (2) ____________________
- Don't know (3)

QID30 Have you specifically sought to include Indigenous Australians in consumer partnerships with your health service?

- Yes (how did you recruit participants?, the box will expand to fit your answer) (1) ____________________
- No (why not? e.g. too difficult; not relevant to the services we offer. The box will expand to fit your answer) (2) ____________________
- Don't know (3)

QID31 Have you specifically sought to include people with different levels of physical or sensory disabilities in consumer partnerships with your health service?

- Yes (how did you recruit participants?, the box will expand to fit your answer) (1) ____________________
- No (why not? e.g. too difficult; not relevant to the services we offer. The box will expand to fit your answer) (2) ____________________
- Don't know (3)

QID32 Have you specifically sought to include people with mental health or psycho-social issues in consumer partnerships with your health service?

- Yes (how did you recruit participants?, the box will expand to fit your answer) (1) ____________________
- No (why not? e.g. too difficult; not relevant to the services we offer. The box will expand to fit your answer) (2) ____________________
- Don't know (3)

QID33 Have you specifically sought to include people with cognitive disabilities unrelated to age (i.e., intellectual disability or acquired brain injury) in consumer partnerships with your health service?

- Yes (how did you recruit participants?, the box will expand to fit your answer) (1) ____________________
- No (why not? e.g. too difficult; not relevant to the services we offer. The box will expand to fit your answer) (2) ____________________
- Don't know (3)

QID34 Have you specifically sought to include socially disadvantaged people in consumer partnerships with your health service?

- Yes (how did you recruit participants?, the box will expand to fit your answer) (1) ____________________
- No (why not? e.g. too difficult; not relevant to the services we offer. The box will expand to fit your answer) (2) ____________________
- Don't know (3)

QID35 Summarising your partnerships (again only since 2013).

QID36 How highly do you rate your health service in terms of your partnerships with consumers?

- very good (1)
- good (2)
- neither good nor bad (3)
- could be improved a bit (4)
- could be improved a lot (5)
- None of the above (please explain, text box will expand to fit answer) (6) ____________________

QID37 Have you had a formal external assessment of your health service partnerships with consumers e.g. such as hospital accreditation

- Yes (1)
- No (2)
- Don't know (3)

Answer If Have you had a formal external assessment of your health service partnerships with consumers e.g.... Yes Is Selected

QID38 How has an accreditation body /system rated your service on consumer partnership?

- Not met (1)
- satisfactorily met (2)
- met with merit (3)
- have not been assessed (4)
- don't know the outcome (5)
- None of the above (please explain, text box will expand to fit answer) (6) ____________________

QID39  In what type of location is the service based?(if you are unsure, access this link / map) http://www.doctorconnect.gov.au/internet/otd/publishing.nsf/Content/locator

- RA1 - Major cities of Australia (metro) (1)
- RA2 - Inner Regional Australia (2)
- RA3 - Outer Regional Australia (3)
- RA4 - Remote Australia (4)
- RA5 - Very Remote Australia (5)
- Not sure (please provide some detail) (6) ____________________

QID40 Is there anything else you would like to add about consumer partnerships, your health service and how you have responded to this survey?

QID41 Finally, we would really value speaking further with you about your experiences of partnering with consumers. If you are willing to be contacted for a short conversation, please provide a name and contact below:

Name (1)

Health Service (2)

Contact email (3)

Contact phone (4)
